# Modelling of spatial infection spread through heterogeneous population: from lattice to partial differential equation models

**DOI:** 10.1098/rsos.220064

**Published:** 2022-10-05

**Authors:** Arvin Vaziry, T. Kolokolnikov, P. G. Kevrekidis

**Affiliations:** ^1^ Department of Mathematics and Statistics, Dalhousie University Halifax, Nova Scotia, Canada B3H3J5; ^2^ Department of Mathematics and Statistics, University of Massachusetts, Amherst, MA 01003-4515, USA

**Keywords:** epidemic spread, disease modelling, COVID

## Abstract

We present a simple model for the spread of an infection that incorporates spatial variability in population density. Starting from first-principle considerations, we explore how a novel partial differential equation with state-dependent diffusion can be obtained. This model exhibits higher infection rates in the areas of higher population density—a feature that we argue to be consistent with epidemiological observations. The model also exhibits an infection wave, the speed of which varies with population density. In addition, we demonstrate the possibility that an infection can ‘jump’ (i.e. tunnel) across areas of low population density towards areas of high population density. We briefly touch upon the data reported for coronavirus spread in the Canadian province of Nova Scotia as a case example with a number of qualitatively similar features as our model. Lastly, we propose a number of generalizations of the model towards future studies.

## 1. Introduction

In the era of coronavirus, the ongoing public discussion frequently refers to the reproduction number *R*_0_ as a (simple) single-number diagnostic that captures the entire epidemic for a given country or region; for a summary of mathematical discussions of this diagnostic, we refer the interested reader to [1–3]. In reality, *R*_0_ is a parameter that changes locally—a feature that has not only been realized during the COVID-19 pandemic (see, e.g., [4]), but also one that has been well known for similar outbreaks of other diseases such as dengue [5]. For example, it is natural to expect that areas with high population density and/or limited public health measures are hit much harder than more rural areas or regions with strict health controls (masking and distancing). This suggests the limited value of describing the entire population by a single reproduction number *R*_0_. In light of such considerations, herein we are interested in modelling how the spread of disease depends on local spatio-temporal circumstances. There is a growing literature on understanding the effect of geography on the spread of disease [6–8]. One of the key parameters affecting the disease spread is population density. Our aim is thus to develop a simple, potentially generalizable model that captures the effects of population density and local differences on overall epidemic spread.

At the heart of many epidemiology models, and in the frame of this study as well, are the so-called compartmental models, consisting of various classes of individuals and their interactions. Among the many possibilities that have arisen not only in the context of COVID-19 but also earlier, we note the formulation of ordinary differential equation (ODE) models [9–12], statistical models [10,13], stochastic models [14], agent-based models [15,16], spatial network models [13,17] and partial differential equation (PDE) models [18,19]; see also [7,15,20] for reviews. Some of these works turn out to have a very deep influence on public thinking and policy [11,16].

The focus of the present work will be on spatially distributed models exploring the evolution of the infection not only temporally but also spatially. Indeed, such models have a time-honoured history, e.g. in the format of meta-population models [8], and have been extensively used in the context of COVID-19 [8]. Such models have been used for a diverse host of countries including China [21,22] and Spain [23], while a comparison of different models developed, e.g. for the US, can be found in the so-called COVID-19 Forecast Hub.^1^ On the other hand, there are also models that develop a PDE perspective such as [24,25], in addition to earlier work by the present authors such as [18,19] (see also references within these works).

Our aim in the present work is to complement the above approaches by means of a first-principles look into the development of the interaction between the different agents as they move through the spatial domain (and interact with each other). In so doing, we will develop a nonlinear dynamical lattice-based approach, which can then be taken to the continuum limit, to yield a systematic PDE model that is argued to be more suitable towards the modelling of COVID-19, as well as other infectious diseases. Indeed, rather than incorporating standard processes such as diffusion and advection into an ODE SIR-type model, this perspective retrieves a nonlinear variant of diffusion, which seems to be more well-suited to such epidemic settings. Additionally, a key advantage of the present model is that it enables a variety of generalizations to account for effects of longer range interactions (and, of course, additional effects such as those, e.g., of age distribution of the pandemic impact). Such potential extensions will be highlighted along the way. It is also relevant to mention that for reasons of both concreteness and also practicality related to the identifiability of the model [26] (which does not escape us as a central issue and a consistent source of concern about complex models), we opt within the present seed study to focus on the prototypical SIR-type model. Generalizations to more detailed models with a higher number of compartments will be evident, including also in connection to earlier work of some of the authors [18,27].

Our presentation will be structured as follows. In §2, we will present the theoretical formulation of our model (and its potential extensions). In §3, we will use it to explore invasion waves and their respective speed. In §4, the onset of an infection outbreak will be examined. Finally, after briefly touching upon the case example of Nova Scotia in §5, we conclude and present some future challenges in §6.

## 2. Theoretical formulation of the model

We start with an agent-based model, with the aim of deriving a cellular automata model from it, and then consider its continuum limit to obtain a PDE system. A similar procedure was used in [28] to derive a spatio-temporal model of spreading of illegal activity. We assume that individuals can get infected by leaving their home and travelling to new locations. However, they don’t just simply walk at random or diffuse: after going out (e.g. for shopping or work), they return to their original (base) location.

**Figure 1.**
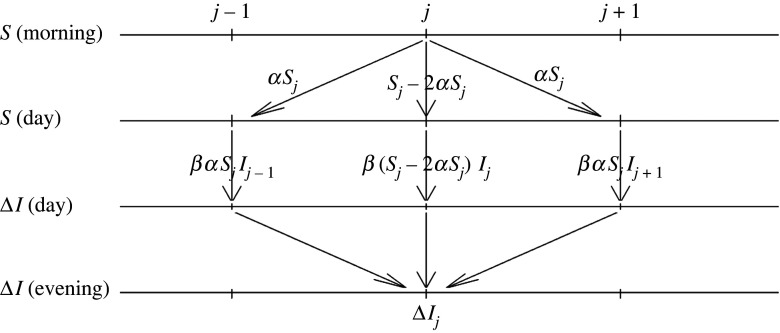
Schematic of the model.

To model individual motion, we discretize the space into bins. Refer to figure 1. For illustration (and although the procedure straightforwardly generalizes to higher dimensions), we assume a one-dimensional grid with bins indexed by *j* = 1 … *N*. Let *S*_*j*_, *I*_*j*_, *R*_*j*_ denote the population of susceptible, infected and recovered in bin *j*. As with the standard SIR model, we assume that infection occurs with some probability *β* per day when a susceptible individual encounters an infected individual. A susceptible individual in bin *j* can get infected in two ways: they either get infected within their own bin (e.g. infection spreading through families at home); or they might leave their home, get infected outside their bin (e.g. going to work, shopping etc.) and then return back to their original location. For simplicity, assume that individuals travel only to neighbouring bins *j* − 1 and *j* + 1 for work/shopping during the day, then return back home in the evening. We will see afterwards how to extend the model past this simplifying assumption. In addition, assume for now that only susceptible individuals can travel (we will deal with a more general case below). Let *α* denote this daily travel rate (so that *αS*_*j*_ susceptibles travel from *j* to *j* + 1 and *αS*_*j*_ travel from *j* to *j* − 1). Let Δ*I*_*j*_ denote new infections per day in bin *j*. With the above assumptions, we obtain

2.1 $$\Delta I_{\;j}=\beta (S_{\;j}-2\alpha S_{\;j})I_{\;j}+\beta \alpha S_{\;j}I_{\;j-1}+\beta \alpha S_{\;j}I_{\;j+1}.$$

Here, *β*(*S*_*j*_ − 2*αS*_*j*_)*I*_*j*_ represents the daily new infections that happen in bin *j*; whereas *βαS*_*j*_*I*_*j*±1_ is the total number of new infections within bin *j* acquired by individuals going to work/shopping etc in the neighbouring bins, then returning home with an infection (due to the interaction of these susceptibles with infected individuals in bins *j* ± 1).

The corresponding SIR model on a lattice then reads

2.2 $$S_{\;j}(t+1)=S_{\;j}-\Delta I_{\;j};\;I_{\;j}(t+1)=I_{\;j}+\Delta I_{\;j}-\gamma I_{\;j}\;\mathrm{and}\;R_{\;j}(t+1)=R_{\;j}+\gamma I_{\;j}.$$

We now consider the continuum limit of this model, in the limit of many bins. Let d*x* be the grid spacing, so that *I*_*j*_ ≈ *I*(*x*) where *x* = *j* d*x*. We then estimate

$$\beta (S_{\;j}-2\alpha S_{\;j})I_{\;j}+\beta \alpha S_{\;j}I_{\;j-1}+\beta \alpha S_{\;j}I_{\;j+1}\approx \beta SI+\beta {\left(dx\right)}^{2}\alpha SI_{xx}$$

and we estimate *S*_*j*_(*t* + 1) − *S*_*j*_(*t*) ≈ *S*_*t*_ (up to a rescaling by the time discretization increment d*t*) and similarly for *I* and *R*. The resulting equations become

2.3 $$S_{t}=-D\beta SI_{xx}-\beta SI,\;I_{t}=D\beta SI_{xx}+\beta SI-\gamma I\;\mathrm{and}\;R_{t}=\gamma I,$$

where

2.4 $$D={\left(dx\right)}^{2}\alpha .$$

Note that unlike many other PDE models [24,25,29,30], the ‘diffusion’ term depends explicitly on the susceptible population density *S*(*x*, *t*). Moreover, the ‘diffusion’ enters into equation for *S* with a *negative* sign, whereas it has a positive sign in the equation for *I*.

Simulations (not shown) of continuum (2.3) and discrete models (2.2) show that they agree well, provided that the changes in both space and time are sufficiently smooth. Note that in the discrete model, 2*α* represents a fraction of individuals ‘going to work’ each day and, as such, we must have *α* < 0.5; otherwise, the discrete model (2.2) becomes unphysical and the solution blows up.

Next, consider a more realistic model, where both susceptible as well as (e.g. asymptomatic [8,27]) infected individuals travel, with rates *α*_*S*_ and *α*_*I*_, respectively.

Then, (2.1) gets replaced with

2.5 $$\begin{array}{rl} \Delta I_{\;j} & =\beta (S_{\;j}-2\alpha_{S}S_{\;j})(I_{\;j}+\alpha_{I}(I_{\;j-1}+I_{\;j+1}-2I_{\;j}))\\ & \;+\beta \alpha_{S}S_{\;j}(I_{\;j-1}+\alpha_{I}(I_{\;j-2}+I_{\;j}-2I_{\;j-1}))\\ & \;+\beta \alpha_{S}S_{\;j}(I_{\;j+1}+\alpha_{I}(I_{\;j+2}+I_{\;j}-2I_{\;j+1})). \end{array}$$

The limiting procedure results in equations (2.3), but with *α* = *α*_*S*_ + *α*_*I*_. Hence, we expect this to be the prototypical PDE-type model within this class of compartmental systems.

The remainder of the paper is concerned with the study of continuum equations (2.3). Before we do so, it is relevant to add a word about the possibility that travelling does not solely occur to bin *j* ± 1 with rate *α* ≡ *α*_1_, but similarly to *j* ± 2 with rate *α*_2_ etc. Then, it is straightforward to show that the Laplacian term is replaced by a nonlocal term of the form S(x)∫K(x−y)I(y) dy, where the (decaying with distance) kernel *K* is proportional to the probability of travelling between locations of distance |*x* − *y*|. A straightforward Taylor expansion around the vanishing argument of the kernel can be used to see that the diffusivity *D* above is proportional to the second moment (i.e. the variance) of the above kernel. More specifically, assuming for simplicity an even (or more generally isotropic) kernel

$$\int K(x-y)I(y)\;dy=\int K(\xi )I(\xi +x)\;d\xi \approx \left(\int K(\xi )\;d\xi \right)I(x)+DI_{xx}+\cdots$$

Accordingly, the first term renormalizes *β*, while the second one produces the diffusive approximation with $D=(1/2)\int K(\xi )\xi^{2}\;d\xi$. We can thus see how such beyond-nearest-neighbour terms can generalize the model, while falling back to it in the simplest diffusive correction level of approximation. Our model also easily generalizes to two dimensions with motion along a two-dimensional grid. In this case, it is easy to see that *I*_*xx*_ in (2.3) get replaced by a two-dimensional Laplacian *I*_*xx*_ + *I*_*yy*_. It is also interesting to further perceive how anisotropic kernels may lead to directed (convective rather than diffusive) motion, although the latter possibility will not be pursued further here.

## 3. Examination of an invasion wave

One of the main effects of introducing a spatial dimension is that the infection typically propagates from its origin. When the movement is sufficiently slow, this propagation happens in a wave-like fashion. One of the, arguably, simplest settings exhibiting wave propagation is the context of the KPP–Fisher equation, modelling propagation of invasive species inside a favourable medium (see, e.g., [31] for a review),

3.1 $$u_{t}=du_{xx}+ru-su^{2}.$$

The travelling-wave solution has the form *u*(*x*, *t*) = *U*(*x* − *ct*), where *U* satisfies the corresponding co-travelling ODE

$$-cU^{\prime }=dU^{\prime \prime }+rU-sU^{2}.$$

We seek a wave propagating from left to right, so that *U*(*z*) → 0 as *z* → +∞, and *U* → *r*/*s* as *z* → −∞. Following the relevant standard theory and linearizing at the front of the wave (*z* → +∞), we can seek a solution of the form

U(z)∼exp⁡(−λz),as z→+∞,

which yields a dispersion relationship between the speed *c* and decay rate *λ* of the form

3.2 $$c=d\lambda +\frac{r}{\lambda }.$$

The minimum speed of propagation is obtained by minimizing (3.2) over all admissible decay rates *λ* > 0, which yields

3.3 $$c_{\min}=2\sqrt{dr}.$$

Numerical experiments confirm that the speed of propagation approaches *c*_min_ for a wide range of initial conditions, so long as *u*(*x*, 0) decays ‘sufficiently fast’ as *x* → ∞. This is a well-known feature of the KPP–Fisher equations [31,32]. Note that this speed only depends on linear terms in (3.1) (i.e. it is independent of the value of *s*). Now, suppose that the parameters *d*, *r* are functions of space *x*. If they vary sufficiently slowly, we expect that the speed of propagation will still be well approximated by (3.3). This is the so-called adiabatic approximation. We now return to the SIR model of equation (2.3). At the front of the infection wave, we estimate *S*(*x*) by *S*_0_(*x*), where *S*_0_(*x*) is the corresponding initial condition. The implicit assumption here is that *I*, *R* ≪ *S* and hence maintaining *S* ≈ *S*_0_ is a reasonable approximation. Then, this leads to the effective linear PDE for *I*(*x*, *t*)

3.4 $$I_{t}∼D\beta S_{0}(x)I_{xx}+(\beta S_{0}(x)-\gamma )I.$$

Assuming that the motion is sufficiently slow (*D* ≪ *O*(1)), we linearize at the front of the wave similarly to our discussion above for the KPP–Fisher equation and obtain the following approximation for the speed of propagation

3.5 $$c(x)∼2\sqrt{D\beta S_{0}(x)(\beta S_{0}(x)-\gamma )}.$$

**Figure 2.**
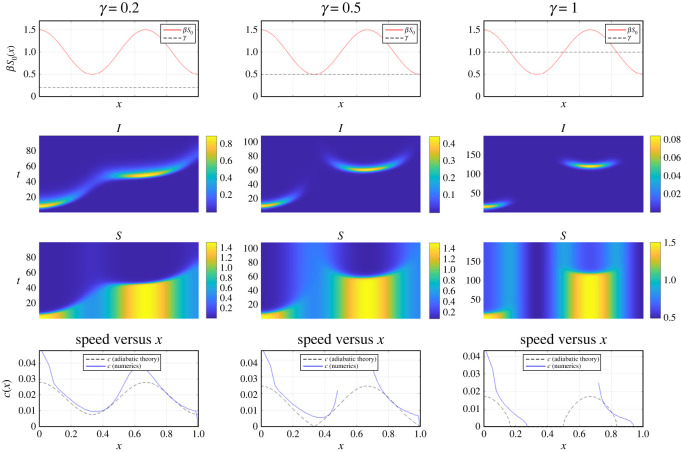
Simulation of an infection wave propagating through a heterogeneous population, for several values of *γ* as indicated. Other parameters are: *β* = 1, *S*_0_ (*x*) = 1 + 0.5cos (3*πx*), *I*_0_(*x*) = 0.01exp (−1000*x*) and *D* = 0.0001. The top row shows *S*_0_ and *γ*. Areas where *βS*_0_(*x*) > *γ* (i.e. where the red solid line is above the dashed line) are favourable for an outbreak. The second row shows *I*(*x*, *t*), the infection density propagating through the population. The third row shows *S*(*x*, *t*), the density of susceptibles. The last row shows the speed *c* of the wave as a function of wave position *x*, comparing numerics with the adiabatic theory. Note how the infection ‘tunnels’ through areas of low infectivity in the last two columns. We used *N* = 200 meshpoints and Δ*t* = 0.001. See Appendix A for Matlab code to simulate (2.3).

Figure 2 shows a comparison between the formula (3.5) and full numerical simulations for several choices of *γ*. We used an implicit–explicit finite difference scheme to simulate the PDE of equation (2.3). The numerical speed *c* is computed by tracking the front of the infection wave *x*(*t*). At any given time *t*, this is done by solving *I*(*x*, *t*) = 0.0001 for *x* = *x*(*t*), and then approximating *c*(*t*) ≈ *x*(*t* + Δ*t*)/Δ*t*. The discontinuity of the blue curve in the second and third columns is due to the fact that *I*(*x*, *t*) dips below 0.0001 before reappearing on the right side. As can be seen in figure 2, the adiabatic approximation (3.5) works relatively well in the areas where *βS*_0_(*x*) − *γ* > 0. The formula breaks down in the areas where *βS*_0_(*x*) − *γ* ≤ 0.

These areas can be thought of as ‘buffer zones’ where effective infection growth is negative; otherwise stated, the local *R*_0_ is below unity and infection is suppressed therein. Nonetheless, the infection wave is able to ‘tunnel through’ these areas, with some delay; see §5 for further investigation of this phenomenon.

## 4. The onset of the outbreak

Note that equations (2.3) admit a ‘trivial’ solution corresponding to no outbreak; namely, *I*(*x*, *t*) = 0 and *S*(*x*, *t*) = *S*_0_(*x*), where *S*_0_(*x*) describes the initial population distribution. We now explore the conditions for the initiation of the outbreak. At the onset of the outbreak, we may assume that *I*(*x*, *t*) ≪ 1. Linearizing equation (2.3), in analogy to what is done for the ODE variant of the model to obtain the bifurcation associated with the spreading of the infection [1–3], leads to an equation for *I* only of the form (3.4). Looking for solutions of the form *I*(*x*, *t*) = e^*λt*^*ϕ*(*x*), we obtain an eigenvalue problem

4.1 $$\frac{\lambda +\gamma }{\beta S_{0}(x)}\phi =D\phi_{xx}+\phi .$$

First, consider the limit *D* = 0. In this case, each point *x* in space evolves separately, and the eigenvalues *λ* are given by *λ* ∼ *βS*_0_(*x*) − *γ*. The outbreak is therefore prevented when *βS*_0_(*x*) < *γ* for all *x*, or *γ* > *γ*_*c*_, where

4.2 $$\gamma_{c}=\beta \max_{x}S_{0}(x).$$

This can be thought of as a ‘spatially extended’ generalization of the ODE result, in that the points in space are practically independent, and hence for the epidemic to be suppressed, this needs to be achieved ‘individually’ for every spatial point.

More generally, we define *γ*_*c*_ to be a threshold value of the decay parameter *γ*, corresponding to the zero-eigenvalue of (4.1). Namely, *γ*_*c*_ satisfies

4.3 $$\frac{\gamma_{c}}{\beta S_{0}(x)}\phi =D\phi_{xx}+\phi ;$$

the outbreak occurs if, and only if, *γ* < *γ*_*c*_. For general *S*_0_(*x*) and *D*, the problem (4.3) does not have an explicit solution. However, we expect *γ*_*c*_ to approach (4.2) as *D* → 0. We now derive the corrections to (4.2) in the limit of small but non-zero *D*, i.e. for 0 < *D* ≪ 1 using asymptotic analysis. We expect the outbreak to first occur near the maximum of *S*_0_. Let *x*_*m*_ be the point at which *S*_0_(*x*) has its maximum. As such, we expand

$$x=x_{m}+\varepsilon y,$$

where *ɛ* is a small constant to be determined. Near *x*_*m*_, write

$$S_{0}(x)∼A(1-B\varepsilon^{2}y^{2})+O(\varepsilon^{3}),\;\text{where }A=S_{0}(x_{m});\;AB=\frac{-S_{0}^{\prime \prime }(x_{m})}{2}$$

and we expand 1/*S*_0_(*x*) ∼ (1 + *Bɛ*^2^*y*^2^)/*A*. Problem (4.3) then becomes

$$\frac{\gamma_{c}}{A\beta }(1+B\varepsilon^{2}y^{2})\phi ∼D\varepsilon^{-2}\phi_{yy}+\phi .$$

We now choose *ɛ* so that *Bɛ*^2^ = *Dɛ*^−2^. In other words, we let

$$\varepsilon \;:\;=D^{1/4}B^{-1/4}.$$

Assuming *ɛ* is small, to leading order we obtain an eigenvalue problem

4.4 $$\phi_{yy}-y^{2}\phi =-\mu \phi ,\;y\in R$$

with

4.5 $$\mu =-\left(\frac{\gamma_{c}}{A\beta }-1\right)D^{-1/2}B^{-1/2}.$$

Equation (4.4) is a well-known quantum-harmonic oscillator eigenvalue problem, where eigenfunctions are given in terms of Hermite polynomials multiplied by a Gaussian. The corresponding eigenvalues are given by

μ=1,3,5,7,…

The smallest eigenvalue is *μ* = 1. Setting *μ* = 1 in (4.5), we obtain the following formula for the threshold value *γ*_*c*_

4.6 $$\frac{\gamma_{c}}{\beta }∼S_{0}(x_{m})-D^{1/2}{\left(\frac{-S^{\prime \prime }(x_{m})}{2}\right)}^{1/2}S_{0}{\left(x_{m}\right)}^{1/2}+O(D).$$

For example, take *S*_0_(*x*) = *a* + sin (*πx*); *β* = 1, *x* ∈ (0, 1). Then, the maximum occurs at *x*_*m*_ = 0.5 and we obtain

4.7 $$\gamma_{c}∼1+a-D^{1/2}\pi {\left(1+a\right)}^{1/2}2^{-1/2}.$$

Table 1 compares the formula (4.7) with the fully numerical solution of the eigenvalue problem (4.3), in the case of *a* = 0. The relative error appears to scale with a direct proportionality to *D*.

**Table 1.** Comparison of asymptotics and numerics.

*D*	0.01	0.005	0.0025	0.00125
*γ*_*c*_ from numerics (4.3)	0.7686	0.8429	0.8889	0.9214
*γ*_*c*_ from asymptotics (4.7)	0.7778	0.8389	0.8871	0.9206
relative error	1.18%	0.47%	0.20%	0.093%

Let us also study the asymptotics in the limit of large *D*, on the domain *x* ∈ [0, *L*] with Neumann boundary conditions *ϕ*(0) = *ϕ*(*L*) = 0. In this case, we expand *ϕ* in (4.3) as

$$\phi =\phi_{0}+\frac{1}{D}\phi_{1}+\cdots$$

At leading order in *D*, we obtain *ϕ*_0*xx*_ = 0. Together with boundary conditions *ϕ*^′^(0) = *ϕ*^′^(*L*) = 0, this yields *ϕ*_0_ (*x*) =const. By scaling, we may then take *ϕ*_0_ = 1. The next-order equation for *ϕ*_1_ then becomes

4.8 $$\frac{\gamma_{c}}{\beta S_{0}(x)}=\phi_{1xx}+1.$$

We then integrate both sides to obtain

4.9 $$\gamma_{c}∼\beta {\left(\frac{1}{L}\int_{0}^{L}\frac{1}{S_{0}(x)}\right)}^{-1},\;D\gg O(1).$$

The quantity ${\left((1/L)\int_{0}^{L}{\left(S_{0}(x)\right)}^{-1}\right)}^{-1}$ is called the harmonic average of *S*_0_(*x*).

For example, take *S*_0_(*x*) = *a* + sin (*πx*) with *x* ∈ (0, 1). Then, (4.8) integrates to

4.10 $$\gamma_{c}∼\left\{ \begin{array}{cc} \frac{\pi \sqrt{1-a^{2}}}{\log\left(1+\sqrt{1-a^{2}}\right)-\log\left(1-\sqrt{1-a^{2}}\right)}, & 0<a<1\\ \frac{\pi }{2}, & a=1\\ \frac{\pi \sqrt{a^{2}-1}}{\pi -2\arctan{\left((a^{2}-1\right)}^{-1/2})}, & a>1. \end{array}\right.$$

**Figure 3.**
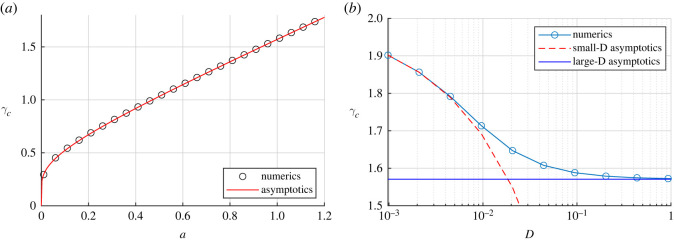
(*a*) Threshold for outbreak *γ*_*c*_ in the limit of ‘large’ *D*. Here, *D* = 1 and *S*_0_(*x*) = *a* + sin (*πx*), *x* ∈ (0, 1); *β* = 1. The numerical solution of (4.3) and asymptotics given by (4.10) are both shown. They are indistinguishable, with relative error less than 0.1%. (*b*) Threshold as a function of *D* with *S*_0_(*x*) = 1 + sin (*πx*). Small and large-*D* asymptotics are also shown.

Figure 3*a* compares the asymptotics (4.10) with full numerical simulations of (4.3) for a wide range of *a*, and with *D* = 1. Despite a relatively small value of *D*, the agreement is excellent over the entire range of *a* (within 0.1%). On the right, we fix *a* = 1 and vary *D*; as can be seen, both large- and small-*D* asymptotics agree very well with full numerics. The intermediate regime of *D*, where neither of our approximations is of value, illustrates the most substantial deviations, yet we still have a very adequate description of the two asymptotic limits.

Finally, note that for constant population density *S*_0_, the threshold *γ*_*c*_ defined by (4.3) is independent of *D*, and both (4.9) and (4.6) yield *γ*_*c*_ = *βS*_0_. This may also be rather natural to expect as in that case, the diffusion term is ‘deactivated’ and we are effectively back to the ODE problem case. One might naively expect that in the large-*D* limit, *S*_0_ would be replaced by the arithmetic average of *S*_0_(*x*). However, our analysis shows that the more appropriate formula is to take a *harmonic* average of *S*_0_(*x*), as in (4.9).

## 5. Indicative observations from COVID-19 in Nova Scotia and ‘tunnelling’

**Figure 4.**
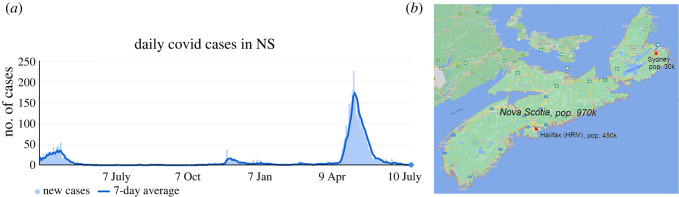
(*a*) Daily COVID-19 cases for the province of Nova Scotia, from May 2020 to July 2021. Vertical axis shows the number of people with a reported positive test on any given day. Source: Google COVID data for NS. Around 80% of the cases occurred in the Halifax Regional Minicipality, which contains about 50% of the population of Nova Scotia. (*b*) Map of Nova Scotia. Sources: Wikipedia.

As a case study, consider the Canadian province of Nova Scotia where some of the authors of this paper reside. It has a population of about 1 million, with slightly less than half of those living in Halifax Regional Minicipality (HRM: the city of Halifax and surrounding area). The second-biggest town is Sydney (see map in figure 4*b*) with a population of 30 000. Much of the rest of the province has relatively low population density. Nova Scotia managed to completely suppress the initial outbreak in the spring of 2020 using very strict stay-at-home orders and border controls. Any visitor required a strict self-isolation quarantine of two weeks upon entry. As a result, there were very few locally transmitted cases up until April 2021; stringent health measures managed to extinguish the few localized outbreaks that did occur before they could spread.

Figure 4 shows the daily COVID case numbers for Nova Scotia. In total, as of July 2021, Nova Scotia had about 5800 cases, which is about 0.6% of the total population of 1 million. About 70% of these cases occurred during the ‘third wave’ in April–June 2021. Very few cases occurred in between the three waves—and most of those were travel-related in quarantine (i.e. not involving community spread). Although less than half of the Nova Scotia population lives in HRM, it was responsible for 79% of the cases overall and 81% of the cases in the third wave. Another 10.5% of cases occurred in Sydney, about 400 km (4.5 h drive) from Halifax. Together, HRM and Sydney were responsible for over 90% of all infections, despite having about half of the overall population of the province. Despite its relatively smaller size, the infection rate in Sydney was about 2.5 times that of Halifax during the third wave.

The main takeaway lesson from this brief data summary, in connection to the qualitative model features discussed herein, is that the rate of infection is much higher in denser urban regions than the rest of Nova Scotia, which is mainly rural with low population density. This is indeed consistent with our model and its corresponding observations. In addition, due to stringent health measures, it is likely that the epidemic in most of the regions of Nova Scotia did not spread—even during the third wave—as almost all infections came from HRM and Sydney—the two biggest population centres in Nova Scotia. Despite strict travel restrictions (even inter-provincial travel was banned during the third wave in May 2021), the infection was able to ‘tunnel through’ the rural areas from HRM to Sydney.^2^

**Figure 5.**
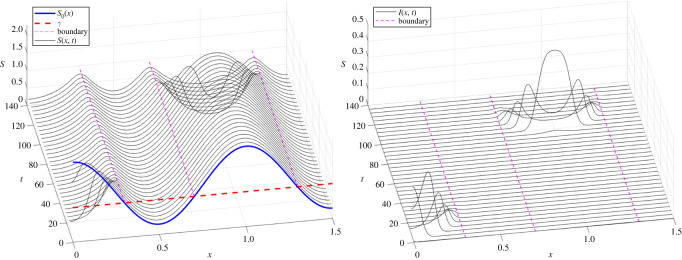
Infection ‘tunnelling’ through a barrier. Initial conditions were taken to be *S*_0_(*x*) = 1.3 + cos (2*πx*), with *γ* = *β* = 1 and x∈[0,1.5]. Without spatial interactions (*D* = 0), the disease is suppressed in the middle region *x* ∈ [0.298, 0.701], as well as for *x* > 1.298. Here, we take *D* = 0.00005. The disease is introduced at *t* = 0 at the left end *x* = 0; corresponding to initial conditions *I*(*x*, 0) = 0.001exp (−1000*x). An infection wave propagating to the right is initially observed but appears to die out around *t* ≈ 30 as it hits the buffer region at *x* ≈ 0.3. However, it is able to ‘tunnel through’ the buffer region, reappearing at *x* = 1 (where *S*_0_ has its maximum) when *t* ≈ 90, then propagating from there to the rest of the infectious region x∈[0.7,1.3].

Motivated by the above observations, we now show that our model can reproduce, at least qualitatively, a ‘tunnelling-through’ effect, where the infection can spread between two regions of locally positive growth, even when separated by a ‘buffer zone’ of negative growth (i.e. infection suppression). Consider a sample simulation as shown in figure 5, with *S*_0_ = *S*_0_(*x*) = 1.3 + cos (2*πx*), with*x* ∈ (0, 1.5) and *β* = *γ* = 1. Locally (in the limit of *D* = 0), the infection is suppressed in the middle region *x* ∈ (0.298, 0.701) as well as for *x* > 1.298 where *S*_0_(*x*)*β* < *γ*, and grows to the left and to the right of that region. We initially introduce the infection near the left boundary of *x* = 0. The outbreak then takes over the entire left region 0 ≤ *x* ≤ 0.298 by the time *t* = 20. Then, for a relatively long time 20 < *t* < 100, nothing appears to happen. But eventually at around *t* ≈ 100, the infection manages to ‘jump’ over to the right region and reappears at *x* = 1 (where *S*_0_(*x*) has its maximum), then spreads from there both to the left and to the right until the entire region 0.701 ≤ *x* ≤ 1.298 is infected.

Qualitatively, the ‘tunnelling’ behaviour can be explained by the presence of diffusion, which allows for an exponentially small amount of infection to ‘diffuse’ within the suppression region *x* ∈ (0.298, 0.701) until it is eventually amplified in the outbreak region *x* > 0.701. Further investigation of this phenomenon is an interesting topic for future work.

It is interesting to note that when the infection reappears at *t* ≈ 100, it does so at *x* = 1 rather than *x* ≈ 0.7. The reason merits further investigation, but roughly speaking, this happens because the local growth rate of infection is given roughly by *S*_0_(*x*)*β* − *γ* and is the highest at the maximum of *S*_0_(*x*). We remark that similar ‘jumps’ of infection were investigated using network models in [33]. There, the authors investigated how network connectivity (and particularly, the presence of a few ‘long’ edges connecting otherwise ‘local’ regions) caused the appearance of new infection clusters. In [34], the authors also showed how tracking new clusters can be used to investigate the origin of the epidemic, and how network connectivity can predict the arrival times at various locations.

## 6. Conclusion and future work

We have presented a model of spatio-temporal infection spread. We have started from a lattice variant of the problem and considered a first-principles inclusion of mobility according to which people move to new, adjacent locations (for work, shopping or other purposes), get infected and return to their base in that new infected state. The model allows for extensions whereby the mobility is to different locations (rather than to adjacent bins) with a kernel that decays over distance. The latter constitutes an interesting variant of the current model relevant to examine in future work. Considering the continuum limit of the proposed lattice system, we obtained a PDE (2.3) with state-dependent diffusion terms. Essentially, the scope of our work is to advocate the relevance of consideration of such terms, in addition to local ones and, arguably, instead of regular diffusion processes in this setting. The key assumption in our modelling is that while individuals move around, they do not diffuse, while infection does. While numerous PDE models exist in epidemiology (see, e.g., [18,19,24,25,29] for a sample), most assume either constant diffusion, or diffusion that is prescribed to be spatially dependent. By contrast, we present a first-principles derivation of equation (2.3) from the underlying cellular automata representation of the basic infection mechanisms. Our model naturally leads to a diffusion that scales with the current number of susceptibles. Note that the phenomena such as tunnelling can also be observed in models without the state-dependent diffusion. However, our model should provide a more qualitatively accurate account of how the infection propagates throughout the domain; in particular, one can expect an additional slowdown of propagation in the areas of lower population density due to state-dependent diffusion.

Introducing a spatial component to a basic SIR model also naturally explains why areas of high population density experience higher infection rates than more rural areas (for related approaches, see, e.g., [9,35]). We also generalized the concept of the reproduction number in this spatially variable setting by deriving an eigenvalue problem (4.1), where the solution describes overall decay or spread of the disease. Importantly, the relevant eigenvalue problem near the maximum of the susceptible population can be approximated by a quantum harmonic oscillator, which allows an approximate analytical expression for the critical clearance rate that would avoid the spreading of infection. We have tested the relevant predictions numerically, finding very good agreement with our theoretical results, where appropriate.

Aside from spatially dependent infection rates, our model demonstrates the difficulty of suppressing the outbreaks. As illustrated in figure 5, the disease can ‘tunnel’ between ‘islands’ of positive growth separated by areas of negative growth (i.e. decay) of the epidemic. A better understanding and more systematic quantification of such phenomena is planned for future work.

There are also numerous additional dimensions in which the present consideration can be extended (both literally and figuratively). Indeed, here we restricted considerations to one-dimensional settings, i.e. ‘geographical corridors’. In line with other works such as [18,24], it is naturally more relevant to explore two-dimensional domains. In addition, it is of substantial interest to consider infections across different age groups. Our considerations herein have assumed that the infectiousness and especially recovery properties of the entire population are the same. However, it is well understood that COVID-19 has a far more severe impact on more senior individuals with a weakened immune system; indeed, this has been the basis for designing relevant non-pharmaceutical intervention strategies [36]. It is then of interest to introduce kernels of interaction across a ‘synthetic dimension’ representing age (in addition to spatial dimensions). There, interactions are predominant along the ‘diagonal’, i.e. for people of the same age group, but there are nontrivial interactions between age groups at some ‘distance’ between them (e.g. parents/grandparents and children/grandchildren); see, e.g., [37]. There, a more complicated non-monotonic kernel of interaction across ages may be relevant to include. These are all interesting possibilities currently under consideration for future work and will be reported accordingly in future publications.

## Data Availability

This article has no additional data.

## Footnotes

1 The relevant website is https://covid19forecasthub.org/doc/ensemble/.

2 It is also interesting to note that there are other significant population centres closer to HRM that *did not* see anything near the size of the outbreak in Sydney. This includes the towns of Truro (pop. 23 000, 1 h drive from Halifax) and New Glasgow (pop. 19 000, 2 h drive from Halifax), which not see any significant outbreaks during the third wave. The outbreak in Sydney started with a hockey game, when kids and families from Halifax visited for a hockey tournament at the onset of the third wave, a potential superspreader event. At the end of the day, our simple model is insufficient to make predictions at such localized detail; many of the outbreaks are driven by random events and the luck of the draw, which our deterministic model is not designed to deal with in this first installment. This is naturally an intriguing challenge for further work.

## Appendix A. Matlab code for model (2.3)

The following Matlab code was used to simulate (2.3). It uses implicit–explicit finite differences, where the Laplacian term is discretized implicitly (for numerical stability, allowing for a relatively large time stepping), whereas nonlinear terms are handled explicitly. Cut-and-paste into Matlab to run.
